# The effect of irradiance on the carbon balance and tissue characteristics of five herbaceous species differing in shade-tolerance

**DOI:** 10.3389/fpls.2014.00012

**Published:** 2014-02-04

**Authors:** Thijs L. Pons, Hendrik Poorter

**Affiliations:** ^1^Plant Ecophysiology, Institute of Environmental Sciences, Utrecht UniversityUtrecht, Netherlands; ^2^IBG-2 Plant Sciences, Forschungszentrum Jülich GmbHJülich, Germany

**Keywords:** construction costs, growth analysis, photosynthesis, root respiration, scaling slope analysis, shoot respiration, whole-plant gas exchange

## Abstract

The carbon balance is defined here as the partitioning of daily whole-plant gross CO_2_ assimilation (*A*) in C available for growth and C required for respiration (*R*). *A* scales positively with growth irradiance and there is evidence for an irradiance dependence of *R* as well. Here we ask if *R* as a fraction of *A* is also irradiance dependent, whether there are systematic differences in C-balance between shade-tolerant and shade-intolerant species, and what the causes could be. Growth, gas exchange, chemical composition and leaf structure were analyzed for two shade-tolerant and three shade-intolerant herbaceous species that were hydroponically grown in a growth room at five irradiances from 20 μmol m^−2^ s^−1^ (1.2 mol m^−2^ day^−1^) to 500 μmol m^−2^ s^−1^ (30 mol m^−2^ day^−1^). Growth analysis showed little difference between species in unit leaf rate (dry mass increase per unit leaf area) at low irradiance, but lower rates for the shade-tolerant species at high irradiance, mainly as a result of their lower light-saturated rate of photosynthesis. This resulted in lower relative growth rates in these conditions. Daily whole-plant *R* scaled with *A* in a very tight manner, giving a remarkably constant *R/A* ratio of around 0.3 for all but the lowest irradiance. Although some shade-intolerant species showed tendencies toward a higher *R/A* and inefficiencies in terms of carbon and nitrogen investment in their leaves, no conclusive evidence was found for systematic differences in C-balance between the shade-tolerant and intolerant species at the lowest irradiance. Leaf tissue of the shade-tolerant species was characterized by high dry matter percentages, C-concentration and construction costs, which could be associated with a better defense in shade environments where leaf longevity matters. We conclude that shade-intolerant species have a competitive advantage at high irradiance due to superior potential growth rates, but that shade-tolerance is not necessarily associated with a better C-balance at low irradiance. Under those conditions tolerance to other stresses is probably more important for the performance of shade-tolerant species.

## Introduction

The rate of photosynthesis typically increases with irradiance, particularly in the lower light range. Plant growth, defined here as the increase in dry mass, can then be expected to increase as well, and this is indeed generally observed (Poorter and van der Werf, 1998). At very low irradiances, as found under a dense leaf canopy, photosynthetic rates are inevitably very low. This does not imply, however, that maximization of gross C-gain and minimization of C-loss is necessarily the best strategy for survival under these conditions. Low light not only strongly limits C-availability for growth but may also critically restrict the energy supply for essential metabolic processes, such as maintenance of cellular gradients or protein turnover. Under these conditions, a proper balance between allocation of C to growth and to respiration may be important for survival, as reserves for storage on the one hand and the generation of metabolic energy on the other may be required to meet the challenges of the stressful shade environment.

The rate of photosynthesis per unit leaf area at the growth irradiance (*A*_growth_) is not the only plant trait that determines the biomass increase per unit biomass present (relative growth rate; RGR). From a C-balance perspective, at least four other plant traits co-determine RGR. These are the leaf area per unit leaf mass (specific leaf area, SLA), the fraction of total plant mass present in leaves (leaf mass fraction, LMF), the fraction of daily gross C-gain that is spent in whole-plant respiration (*R/A*) and the C-concentration of the biomass ([*C*]). In formula:

(1) $$\text{RGR }=\;\left\{A_{\text{growth}}.\left(1-R/A\right).\text{SLA}.\text{LMF}\right\}/\left[C\right]$$

Further explanation of the growth equations is given in Appendix 1 and of abbreviations and symbols in Table 1. Each of the variables in equation 1 can be irradiance-dependent. The SLA decreases strongly with increasing irradiance in most plants, which is the result of both an increasing leaf thickness and leaf tissue density (Poorter et al., 2009). This is associated with a higher photosynthetic capacity per unit leaf area. The LMF generally does not change much with irradiance (Poorter et al., 2012b). Variation in [*C*] with growth irradiance is also small, as was shown for leaves at different positions in tree crowns and for whole plants in a shading experiment (Niinemets, 1999; Poorter et al., 2006; Petritan et al., 2010). Additional components, such as losses through tissue death, exudation and volatilization, are quantitatively of little importance in most juvenile plants and are ignored in this study.

**Table 1:** **Abbreviations and symbols, definitions of the variables and units used in this paper**.

**Abbreviations and symbols**	**Explanation**	**Units**
A	Rate of CO_2_ assimilation or photosynthesis	
*A*_a_	*A* per unit leaf area	μmol m^−2^ s^−1^
*A*_growth_	*A* at the growth irradiance, generally per unit leaf area	μmol m^−2^ s^−1^
*A*_sat_	*A* per unit leaf area at light saturation at the leaf level	μmol m^−2^ s^−1^
*A*_m_	*A* per unit dry mass	nmol g^−1^ s^−1^
[*C*]	Carbon concentration; C per unit dry mass	mol g^−1^
CC	Construction costs; glucose required to synthesize a unit of dry matter	g g^−1^
CUE	Carbon use efficiency; the fraction of assimilated carbon invested in growth (CUE = 1 − *R/A*)	
GRC	Growth response coefficient	
LAR	Leaf area ratio; leaf area per unit plant dry mass	m^2^ kg^−1^
LMA	Leaf dry mass per unit leaf area (LMA = 1/SLA)	g m^−2^
LMF	Leaf mass fraction; leaf dry mass per unit plant dry mass	g g^−1^
*N*_a_	Leaf nitrogen per unit leaf area	mmol m^−2^
*N*_m_	Nitrogen per unit dry mass in plant tissue	mg g^−1^
NAR_ge_	Daily net assimilation rate calculated from gas exchange	g m^−2^ day^−1^
PPFD	Photon flux density, restricted to photosynthetically active radiation	μmol m^−2^ s^−1^
PNUE	Photosynthetic nitrogen use efficiency; *A* per unit N	mmol mol^−1^ s^−1^
*R*	Rate of respiration, measured as CO_2_ release or O_2_ consumption	
*R*_m_	*R* per unit dry mass	nmol g^−1^ s^−1^
*R*_a_	*R* per unit leaf area	μmol m^−2^ s^−1^
*R/A*	*R* as a fraction of gross *A*, mostly for whole plants at a daily (24 h) basis	mol mol^−1^
RGR	Relative growth rate; dry mass increment per unit dry mass and time	mg g^−1^ day^−1^
RMF	Root mass fraction; root dry mass per unit plant dry mass	g g^−1^
SLA	Specific leaf area; leaf area per unit leaf dry mass (SLA = 1/LMA)	m^2^ kg^−1^
SMF	Stem mass fraction; stem plus petiole dry mass per unit plant dry mass	g g^−1^
ULR	Unit leaf rate; dry mass increment per unit leaf area and time	g m^−2^ day^−1^

The fourth component in equation 1 is the R/A ratio. Whole-plant *R* can conceptually be divided into *R* associated with growth—and thus processes such as ion uptake and synthesis of new compounds of biomass—and *R* associated with maintenance, which includes turnover of cellular compounds and maintenance of solute gradients (Amthor, 2000). Growth *R* is thus likely to diminish at low irradiance because of a reduced growth rate. However, the same does not necessarily apply to maintenance *R*. If we assume maintenance *R* to be constant, because the associated cellular processes are not affected by light, it would follow that total *R* diminishes less with decreasing growth irradiance as compared to *A*, with a higher *R/A* ratio at low irradiance as a consequence. In juvenile herbaceous plants growing in optimal conditions *R* integrated over 24 h is circa one third of daily *A* (Poorter, 2002). The few data available on the growth irradiance effect on *R/A* show rather constant values, notwithstanding that *A* increases strongly with irradiance (McCree and Troughton, 1966; Poorter, 2002). However, the data are very limited and this hampers a more generalized picture.

Shade-tolerant species are adapted to conditions where net C-gain is typically low. It could therefore be expected that, compared with shade-intolerant species, their C-balance is more favorable at low irradiance. Comparisons of the C-balance between shade-tolerant and intolerant species have been made at the leaf level (e.g., Noguchi et al., 1996, 2005; Lusk, 2002; Craine and Reich, 2005). These studies indicate that there are no differences in gross photosynthesis in these conditions. There is, however, some evidence for a lower leaf *R* and thus lower light compensation points for leaves of shade-tolerant species, but differences are small and not always consistent (Walters and Reich, 1999). However, rather than the leaf-level it is the C-balance at the whole-plant level that counts (Givnish, 1988). Differences between shade-tolerant and intolerant species at the level of whole-plant gas exchange at low irradiance have not been systematically investigated. The question thus remains whether shade-tolerant species have a superior C-balance in shade.

Our study aims for a better understanding of how the C-balance of plants depends on irradiance. An experiment was carried out where plants were grown at different daily irradiances representative of the full range from dense canopy shade to full daylight. First, we establish the basis for further analysis by determining the RGR and its underlying variables through classical growth analysis. Second, we address the question to what extent the components of the C-balance change with the growth irradiance. The evidence presented above suggests a rather constant daily whole-plant *R* as a fraction of photosynthetic C-gain (*R/A*). However, we hypothesize that the *R/A* should increase when *A* decreases to very low values at low irradiance. A third question we analyze is whether there are species-specific differences in the irradiance dependence of the C-balance and its components between shade-tolerant and intolerant species, because we hypothesize that shade-tolerant species may maintain a more favorable C-balance at low irradiance. Although the number of species included is not sufficient for broad generalizations, the comparison of the five species should give indications of such differences. We furthermore address the question what the causes of the possible dependence of the C-balance on irradiance and shade-tolerance could be. For that purpose we measured leaf-level photosynthesis, leaf structure and aspects of the chemical composition.

## Materials and methods

### Plant material and experimental design

The experiments were carried out with juvenile plants of five herbaceous eudicotyledonous species, two shade-tolerant (*Geum urbanum* L. and *Impatiens parviflora* DC.), and three shade-intolerant species (*Chenopodium album* L., *Helianthus annuus* L. and *Rumex palustris* Sm.) Seeds were collected in their natural habitat in the vicinity of Utrecht, except for *Rumex*, which was collected in a floodplain of the river Waal near Nijmegen and *Helianthus*, which was obtained commercially. *Impatiens* seeds were stratified at 4°C for 2 months. Seeds were germinated on sand in the growth room at an irradiance of 200 μmol m^−2^ s^−1^. When the first true leaves were formed, the plants were transferred to 33 L containers with an aerated nutrient solution, having a concentration of 2 mM NO^−^_3_ and other nutrients in proportion as in Hoagland and Snijder (1933). The pH was adjusted regularly at 5.6 and the solution was changed weekly. Conditions in the growth room were a constant air temperature of 20°C, a relative air humidity 70% and a photoperiod of 16 h. Five levels of irradiance (provided by Philips HPI 400 W lamps) were achieved by creating compartments with reflective walls and neutral shade screen on top. The lower part of each compartment was largely open for ventilation. Irradiance levels were ~20, 50, 100, 220, and 500 μmol m^−2^ s^−1^ photosynthetically active radiation, equivalent to daily irradiances of 1.2, 2.9, 5.8, 12.7, and 28.8 mol m^−2^ day^−1^, respectively. Irradiance was checked regularly during growth of the plants.

### Destructive harvesting

To minimize the effect of plant-to-plant variation on the growth parameter estimates, plants of each treatment and species were divided by eye in two size groups before each harvest (Poorter, 1989). Two plants from each size class were then randomly sampled at each of three harvest occasions. The experiment was repeated once and the data were combined, thus resulting in eight plants per harvest, light condition and species, 600 plants in total. Each of the sampled plants was divided into leaf blades, stems plus petioles and roots, after which leaf area and organ fresh mass were determined. Dry mass was measured after drying at 70°C for 48 h. The first harvest was at a whole-plant fresh mass of about 1.5 g, but tended to be somewhat lower at low irradiance due to low initial growth rates and somewhat higher for the large-seeded *Helianthus*. In order to further minimize size differences over the harvest intervals, time until the third harvest varied between 7 and 20 days, depending on growth rate.

### Gas exchange measurements

Right before the second harvest, plants were measured for their rate of net photosynthesis and the respiration of shoots and roots. The plants were taken from the growth room, their above-ground parts were enclosed in gas exchange cuvettes and measured for CO_2_ uptake at the growth irradiance after reaching steady state (*A*_growth_). After 2 h in the dark, the CO_2_ release of the shoots was measured (shoot *R*). Thereafter, the roots were detached, enclosed in light-tight cuvettes with an oxygen electrode for the measurement of O_2_ consumption in water (root *R*). Details of the technique are given by Poorter et al. (1990). A short description is given below.

In the CO_2_ exchange setup where *A*_growth_ and shoot *R* were measured, light was provided by similar lamps as in the growth chamber. Measurement irradiance and temperature were made identical to the growth conditions. CO_2_ concentration in the incoming air was maintained at 400 μmol mol^−1^, and water vapor partial pressure was set at 1200 Pa. CO_2_ uptake and transpiration modified plant cuvette values to a CO_2_ concentration of ~380 μmol mol^−1^ and a relative humidity of circa 65%. The difference in CO_2_ and H_2_O concentration between inlet and outlet air was measured with an IRGA (Licor—6262, Lincoln, NE, USA).

For the measurement of root respiration, fresh nutrient solution was equilibrated with air at the measurement temperature of 20°C. The full root system was detached from the shoots and enclosed in a custom-made air-tight cuvette completely filled with nutrient solution. The rate of decrease of the O_2_ concentration was measured over a period of 15 min with a Clark electrode (Yellow Group Instruments, OH, USA). Due to the small root systems of the plants from the lowest irradiances, the resolution was insufficient for precise measurements. In those cases roots of two individuals were combined for better sensitivity. Unforeseen technical problems with the system caused that only for two species reliable root respiration measurements are available for the full range of growth irradiances.

For separate plants from each species and irradiance treatment, leaf-level gas exchange was measured on recently matured leaves (*n* = 3). Leaves were enclosed in leaf chambers and measurements were done at growth irradiance and at light-saturation. The setup for these leaf-level measurements has been described by Pons and Welschen (2002).

### Chemical analysis

Chemical composition of dry matter was measured on two independent bulk samples per light level and species, for leaf blade material and the rest of the plants separately. C and N concentrations were measured with an elemental analyzer (Carlo Erba, Milan, Italy). Nitrate was determined colorimetrically after extraction with boiling water, and mineral content determined in ash in combination with ash alkalinity. A full description of procedures and calculations is given at Prometheus wiki (http://prometheuswiki.publish.csiro.au).

### Calculations

Growth parameters were calculated as follows. For each of the plants harvested we kept track of whether they were a-priori classified into the “small or large” group. From each category within each experiment we randomly linked one plant of a given harvest to a randomly chosen plant of the other two harvests, giving eight time-series represented by triplets of plants. For each of these time series a linear regression of log_e_-transformed dry masses over time was calculated, with the slope being the RGR. Morphological (SLA, LAR), allocation (LMF, SMF, RMF) and tissue density variables were calculated as average values per triplet. Finally, for each triplet ULR was calculated from RGR/LAR. Each of the eight growth parameter values was thus based on information from an independent set of plants.

The growth response coefficients (GRCs) of components of the RGR summarize the contribution of the variation in the respective growth parameters of equation 1 to the variation in RGR. To this end, a scaling slope analysis was carried out by calculating the regression coefficients of log-transformed growth parameter data versus log-transformed RGR (Poorter and van der Werf, 1998; Renton and Poorter, 2011). The same analysis was done for the ULR and its components, and the contribution of leaf density and leaf thickness to the increase of LMA with irradiance.

*A*_growth_ was calculated from the CO_2_ uptake in the light, and shoot *R* from the CO_2_ release in the dark. Values were expressed per unit leaf area and dry mass as appropriate. Root *R* was calculated from root O_2_ consumption. For the calculation of whole-plant *R* in CO_2_ units, we assumed a respiratory quotient of 1.2, as is often the case in nitrate-fed plants (Poorter et al., 1990). Whole-plant *R/A*, integrated over 24 h, was calculated on the basis of gross photosynthesis by adding shoot *R* to measured shoot net *A*, assuming identical *R* in light and dark. Hence, the *R* in *R/A* refers to 24 h of whole-plant *R* and the *A* to 16 h shoot gross photosynthesis, both calculated on the same expression basis.

A Two-Way ANOVA was performed on log_e_-transformed data using the aov procedure in R (R Core Team, 2013), with Species and Irradiance as main factors. Specific a-priori contrasts between the shade-tolerant and shade-intolerant species were made for the main effect Species, as well as for the Species × Irradiance interaction.

## Results

### Growth analysis

**Figure 1:**
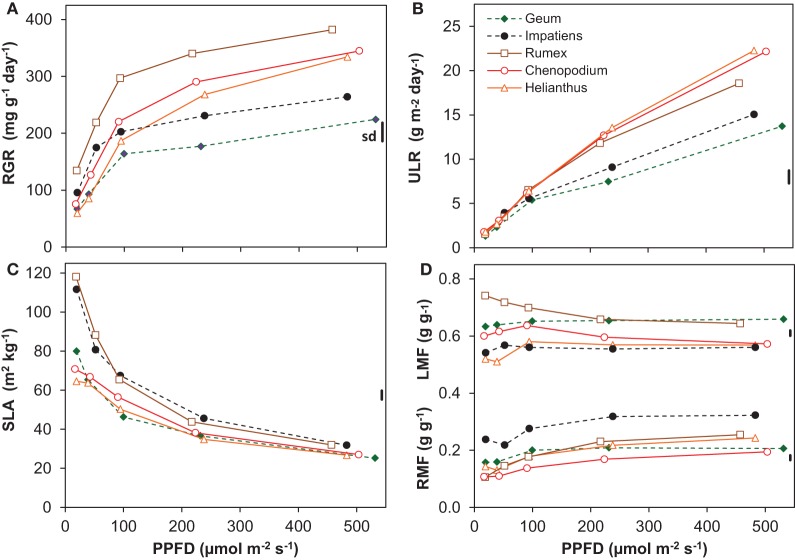
**Growth variables of five species grown at five irradiance levels (Photosynthetic photon Flux Density; PPFD). (A)** Relative Growth Rate (RGR, dry mass increase per unit plant mass and time); **(B)** Unit Leaf Rate (ULR, dry mass increase per unit leaf area and time); **(C)** Specific Leaf Area (SLA, leaf area per unit leaf dry mass); **(D)** Leaf Mass Fraction (LMF, leaf mass per unit plant mass) and Root Mass Fraction (RMF). Means are shown (*n* = 8). The vertical bars at the right of each panel show the common standard deviation (sd), which was calculated as the mean coefficient of variation multiplied by the overall mean and positioned in the panel at the overall mean value of that variable. Continuous lines with open symbols indicate shade-intolerant species, broken lines with closed symbols shade-tolerant ones.

Light had a strong and statistically highly significant effect on most of the growth variables (Figure 1, Table 2). All species had a positive RGR even at the lowest irradiance (Figure 1A), and therefore a positive C-balance. The increase in RGR with irradiance was strong in the lower light range and much less in the higher range. Interestingly, as far as there were differences in RGR between species at the low-irradiance range, there was no clear association with shade-tolerance. However, above an irradiance of ~100 μmol m^−2^ s^−1^ the two shade-tolerant species increased RGR substantially less compared to the intolerant ones (Figure 1A, Table 2). This resulted at the highest irradiance in RGR's of on average 244 and 354 mg g^−1^ day^−1^ for the shade-tolerant and intolerant species, respectively. The ULR across the low irradiance range (20–100 μmol m^−2^ s^−1^) was very similar for all species (Figure 1B). For the shade-intolerant species, ULR increased in an almost linear fashion with irradiance. For the shade-tolerant ones, the ULR increase was less strong, explaining to a large extent their lower RGR under these conditions.

**Table 2:** **Two-Way ANOVA for the variables shown in the figures**.

**Variable**	**Species**	**PPFD**	**Spec X PPFD**	**Tol vs. intol**	**Tol vs.-intol X PPFD**	**Total *df***	***r*^2^**
Figure 1							
RGR	18^***^	76^***^	6^***^	24^***^	26^***^	199	0.89
ULR	2^***^	96^***^	2^***^	71^***^	100^***^	199	0.94
SLA	10^***^	87^***^	2^***^	1^*^	23^***^	199	0.96
LMF	84^***^	2^***^	14^***^	1^***^	15^***^	199	0.88
RMF	47^***^	46^***^	7^***^	53^***^	60^***^	199	0.92
Figure 2							
LMA	9^***^	88^***^	3^***^	0^ns^	21^***^	199	0.94
Leaf density	73^***^	26^***^	1^***^	55^***^	45^***^	199	0.98
Leaf thickness	67^***^	31^***^	2^***^	86^***^	10^+^	199	0.96
Figure 3							
[*C*] (plant)	59^***^	34^***^	6^**^	91^***^	11^ns^	49	0.94
CC (plant)	65^***^	29^***^	6^+^	90^***^	10^ns^	49	0.92
Figure 4							
*A*_*growth*_ (plant)	4^***^	89^***^	6^***^	1^***^	2^***^	195	0.95
*A*_sat_ (leaf)	21^***^	76^***^	3^***^			68	0.97
Figure 5							
*A*_m_ (shoot)	14^***^	79^***^	8^***^	4^**^	38^***^	195	0.91
*R*_m_ (shoot)	13^***^	78^***^	9^***^	0^ns^	35^***^	194	0.88
*R*_m_ (root)	3^+^	89^***^	8^+^			60	0.62
Inst. *R/A* (shoot)	21^***^	63^***^	16^***^	29^***^	63^***^	194	0.66
Daily *R/A* (calc.)	14^***^	33^***^	53^***^	5^ns^	23^**^	194	0.38
Daily *R/A* (meas.)	7^*^	79^***^	14^*^			60	0.57

The 1st, 2nd, and 3rd columns after the variable names show the percentages of explained variance that is due to the effect of Species, growth irradiance (PPFD) and the interaction of the two. The 4th and 5th column show the explained variance of the a-priori contrasts of shade-tolerant (Tol) vs shade-intolerant (Intol) species for the Species main effect and the Species × PPFD interaction. The percentages refer to explained variance relative to the Species and Species × PPFD effect, respectively. The last two columns indicate the degrees of freedom and the adjusted r^2^ of the full model. Significance levels: ns, non-significant; ^+^0.05 < P < 0.10;

* P < 0.05;

** P < 0.01;

*** P < 0.001. The effects that absorb more than 50% of the sum of squares explained by the main and interaction effects of the model are indicated in bold. Note that the root R_m_ and the measured daily R/A whole-plant values are based on Helianthus and Geum only, and that the contrasts in shade tolerance were not tested for A_sat_ measured at the leaf level (leaf) because the data for Geum were not complete.

All plants showed a decrease of SLA with irradiance, strongly in the lower and less so in the higher range (Figure 1C). Two of the shade-intolerant species, *Helianthus* and *Chenopodium*, deviated in the lower light range from the general trend because they did not develop a high SLA there (Figure 1C). The decrease of SLA with irradiance counteracted the almost linear increase of the ULR (Figure 1B), resulting in a curvilinearly increasing RGR. In contrast, the LMF, which is the other morphological component that determines RGR (equation 1, Appendix 1), remained relatively constant (Figure 1D). The exception was *Rumex*, which showed a decreasing trend of LMF with irradiance. All species showed a moderate increase in RMF (Figure 1D) and a decrease in SMF (Supplement Table S1) with irradiance.

### Tissue structure and chemistry

**Figure 2:**
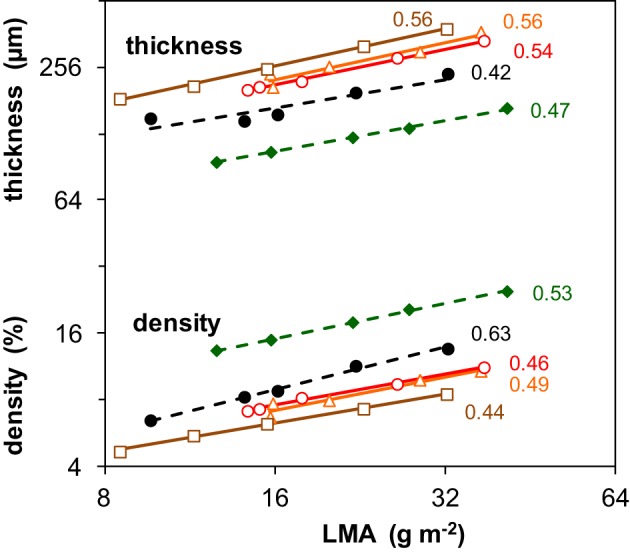
**Changes in leaf tissue density and thickness associated with the changes in leaf dry mass per area (LMA) as induced by different irradiance levels**. Data are given for five species and are average values pertaining to all leaves of the plant. The regression coefficients from a scaling slope analysis, quantifying the fractional contribution of leaf density and thickness to variation in LMA given to the right of the lines. LMA is the inverse of SLA shown in Figure 1C. Mean values (*n* = 8) of density and thickness were log-transformed and plotted against log-transformed LMA. For symbols see Figure 1. The values of LMA, tissue density and leaf thickness are given in Supplement Table S1. For further explanation of the scaling slope analysis see text.

A further analysis of traits underlying SLA can best be done using its inverse, leaf dry mass per unit area (LMA = 1/SLA) (Supplement Table S1). LMA can be factorized as the product of leaf thickness and leaf tissue density, for which we used as proxies fresh mass per area and dry mass per fresh mass, respectively. The importance of light-driven variation in leaf thickness and density for variation in LMA is summarized by the numbers shown in Figure 2. Given that the vales of these scaling slope analyses all centered around 0.5, we concluded that both components were on average equally responsible for the increase of LMA with irradiance. At a given LMA, the leaves of the two shade-tolerant species *Geum* and *Impatiens* were relatively thin and had a higher density compared to the three shade-intolerant species. For these two species, the contribution of tissue density to the irradiance effect on LMA was also somewhat larger (Figure 2). The density of stem and/or petiole tissue also increased with irradiance. However, this was not clearly the case for the density of root tissue (Supplement Table S1).

**Figure 3:**
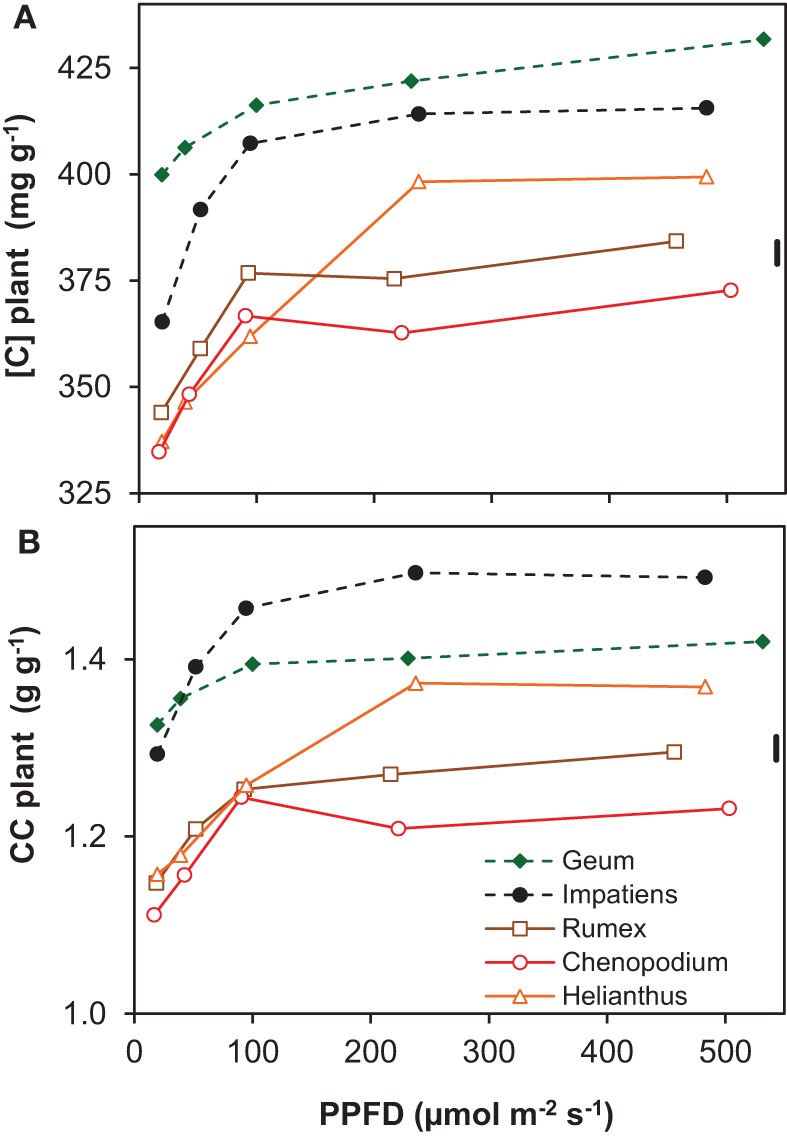
**Chemical composition of whole-plant dry matter of five species grown at five irradiance levels (PPFD). (A)** Carbon concentration in whole-plant dry matter ([*C*]); **(B)** construction costs (CC) of whole-plant dry matter calculated from the chemical composition. Means (*n* = 2 bulk samples) and the common standard deviation (sd) are shown. The chemical composition from which the CC was derived is shown in Supplement Table S2.

The concentration of organic N in leaf dry matter (*N*_m_) was on average 48 mg g^−1^ and generally not much different between growth irradiances (Supplement Table S2). The N content per leaf area (*N*_a_), however, increased almost linearly as a result of the similar increase in LMA (Supplement Tables S1, S3). Remarkable is the low *N*_a_ of *Geum*, particularly at high irradiance, which was caused by its low *N*_m_. The C-concentration in plant dry matter ([*C*]) increased with irradiance and was consistently higher for the shade-tolerant species (Figure 3A). The increase in tissue density with irradiance and shade-tolerance was thus associated with increases in [*C*] (Figures 2, 3A).

### Photosynthesis and respiration

**Figure 4:**
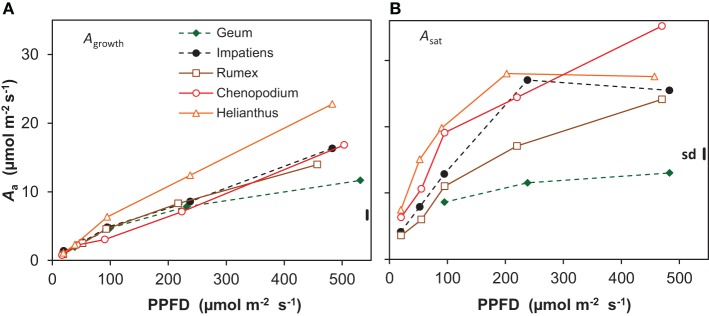
**Photosynthesis per unit leaf area (*A*_*a*_) of five species grown at five irradiance levels (PPFD). (A)** Net photosynthesis per unit leaf area of whole shoots measured at growth irradiance (*A*_growth_); **(B)** light-saturated rate of photosynthesis per unit leaf area (*A*_sat_) measured on recently matured leaves. Means (*n* = 8 and *n* = 3 for *A*_growth_ and A_sat_, respectively) and the common standard deviation (sd) are shown.

Whole-plant photosynthesis measured at growth irradiance and expressed per unit leaf area (*A*_growth_) increased almost linearly with increasing irradiance from an average across all species of 1 μmol m^−2^ s^−1^ at the lowest irradiance to 16 μmol m^−2^ s^−1^ at the highest (Figure 4A). At the highest irradiance, there was substantial variation between the species, with *Geum* showing the lowest value (12 μmol m^−2^ s^−1^) and *Helianthus* the highest (23 μmol m^−2^ s^−1^). Photosynthetic capacity, measured at the leaf level as the light-saturated rate of photosynthesis per unit leaf area (*A*_sat_) also increased with growth irradiance (Figure 4B). *A*_sat_ was higher than *A*_growth_ at all light levels, increasing from on average 5 to 25 μmol m^−2^ s^−1^. Only *Geum* grown at the highest irradiance had similar values for *A*_sat_ and *A*_growth_ (12 μmol m^−2^ s^−1^; Figure 4).

**Figure 5:**
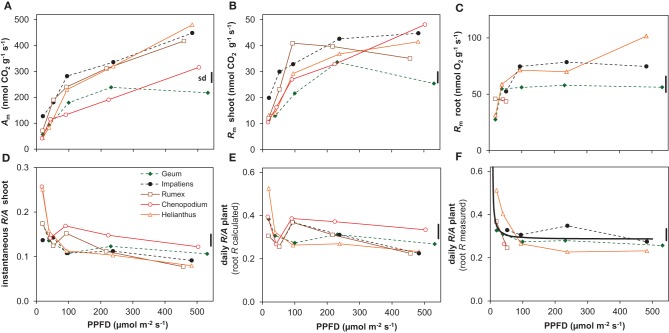
**Photosynthesis (*A*) and respiration (*R*) and the ratio between the two (*R/A*) of the five species grown at different irradiances (PPFD). (A)** Rates of net CO_2_ assimilation at the growth irradiance of whole shoots per unit shoot dry mass (*A*_m_); **(B)** release of CO_2_ in darkness of whole shoots per unit shoot dry mass (dark respiration; *R*_m_); **(C)** root respiration measured as the rate of O_2_ consumption per unit root dry mass (*R*_m_); **(D)** shoot *R/A* ratio on an instantaneous basis; **(E)** whole-plant daily *R/A* ratio using root *R* calculated from shoot *R* (root *R* = 2.9 × shoot *R*); **(F)** whole-plant daily *R/A* ratio based on measured root *R* for the species and irradiances for which root *R* was available. Shown are the means (*n* = 8) and the average standard deviation (sd). The solid line in **(F)** depicts the simulated *R/A* ratio as explained in the text.

*A*_growth_ expressed per unit shoot dry mass (*A*_m_) showed a qualitatively similar curvilinear response to irradiance as RGR, with a strong increase in the lower range but less so in the higher range (Figure 5A). Shoot *R*_m_ also increased with irradiance and was on average 17% of shoot *A*_m_ (Figures 5A,B). However, the relative increase in shoot *R*_m_ between the lowest and the highest irradiance was only about 3-fold, whereas shoot *A*_m_ increased about 6-fold (Figures 5A,B). Consequently, the instantaneous shoot *R/A* ratio decreased with increasing irradiance (Figure 5D).

Root *R*_m_ was successfully measured across the full range of growth irradiances for *Helianthus* and *Geum* only. For the other species data are available for just part of the light range. Averaged over species and conditions, root *R*_m_ was on average 2.9 times higher than shoot *R*_m_. *Helianthus* showed a gradual increase of root *R*_m_ with irradiance, but *Geum*, and also *Impatiens* for which data are available for most of the range, were rather constant across most of the light range, with lower values at the lowest irradiance only (Figure 5C).

Daily whole-plant *R/A* of *Helianthus* and *Geum*, for which the *R*_m_ data are available for the full range, showed a tendency to increase with decreasing irradiance. However, there was a significant difference in irradiance dependence between the two species (Table 2). In *Helianthus R/A* increased relatively strongly below an irradiance of 100 μmol m^−2^ s^−1^, to 0.51 at 20 μmol m^−2^ s^−1^, whereas *Geum* showed a more stable daily *R/A*, increasing to only 0.33 at 20 μmol m^−2^ s^−1^ (Figure 5F). Taking also the available data for the other species into account, the evidence points to a rather stable *R/A* of about 0.3 on average across a large part of the irradiance gradient with a tendency to increase only at the lowest irradiance (Figure 5E). In relative terms, the C-balance was therefore essentially constant across a large part of the growth irradiance gradient (Figure 5F). In absolute terms, however, the C-balance showed an increasing positive difference between *A* and *R* with irradiance.

## Discussion

### Growth at higher irradiances

For the five species investigated in this experiment, the general form of the irradiance response for RGR and its components was similar to what has been reviewed in for example Poorter and van der Werf (1998), with the exception that RGR did not fully saturate at 20 mol m^−2^ day^−1^. The latter phenomenon is often reported for shade-tolerant species, such as *Impatiens* (Evans and Hughes, 1961; Corré, 1983a) and *Geum* (Blackman and Wilson, 1951; Corré, 1983a). In the high-light range shade-tolerant species may even show decreases in RGR (Huxley, 1967; Veenendaal et al., 1996; Poorter, 1999). Growth experiments in an irradiance gradient generate large differences in demand for water and nutrients (Poorter et al., 2012a). A lack of increase or even a decrease in RGR at the high irradiance end in pot-grown plants could therefore be a secondary effect of limitations in the root environment, rather than a result of an intrinsic character of the species. Indeed, a saturated RGR across the high irradiance range was also observed for *Geum* in an outdoor experiment where watering of the plants was not optimal, whereas replication of the experiment with ample water supply resulted in an increasing RGR up to full daylight (Pons, 1977a), similar to what was found here (Figure 1A).

**Figure 6:**
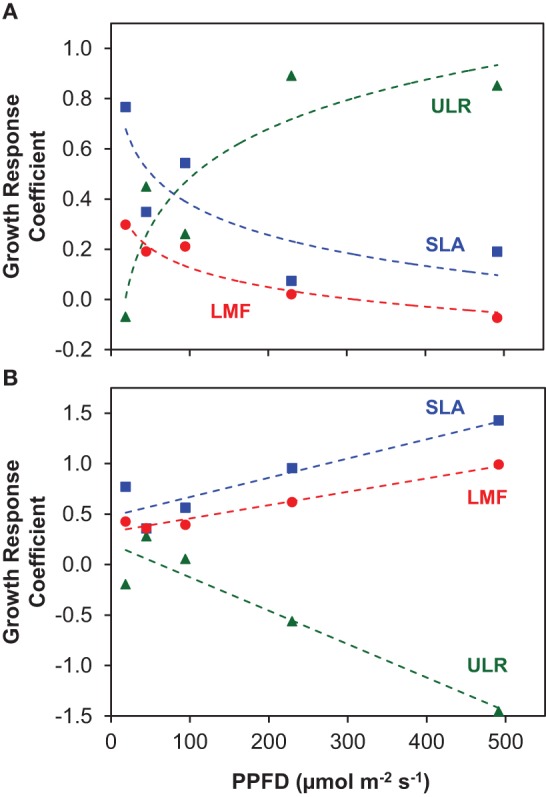
**The Growth Response Coefficients (GRC), showing the contribution of variation in the components ULR, SLA and LMF to variation in RGR for interspecific comparisons at each growth irradiance (PPFD). (A)** All species included in the analysis; **(B)** analysis for the three shade-intolerant species only. The regression coefficients of log-transformed ULR, SLA and LMF on log-transformed RGR at each growth irradiance represent the GRC's.

In their meta-analysis, Poorter and van der Werf (1998) showed a predominance of SLA in explaining species specific variation in RGR for herbaceous plants, and this was also the case among the three shade-intolerant species, with *Rumex* showing the fastest growth and highest SLA at each irradiance level (Figures 1A,C). However, at higher irradiances the result is strongly different when we include the shade-tolerant species: in that case, variation in ULR is the dominant variable that scales with interspecific variation in RGR. The evidence is summarized in Figure 6A, where we calculated the so-called GRC. These GRC values indicate how much the variation in RGR scales with variation in the multiplicative components of equation 2 in Appendix 1 (Renton and Poorter, 2011). They can be used to summarize in a highly efficient way what the relative importance is for each of the components in causing the variation in RGR. A GRC value for ULR of 0, for example, would indicate RGR increases without a concomitant increase in ULR, whereas a value of 1 would imply that a 10% increase in RGR goes with a 10% increase in ULR. In our experiment, the GRC values for SLA and LMF decrease with increasing irradiance, whereas the GRC for the ULR rises from 0 to 0.8, indicating that the higher the irradiance, the stronger interspecific variation in RGR is determined by ULR differences. A similar response was found in the meta-analysis of Shipley (2006), who included both herbaceous and woody species, of which several were shade-tolerant. In other studies with herbaceous species—using partly the same shade-tolerant species as the present experiment—a lower ULR in conjunction with a lower RGR compared to their shade-intolerant counterparts was also found (Pons, 1977a; Corré, 1983a). Similar results were reported for tropical trees, where the differences in RGR at higher irradiances were due to the low ULR of shade-tolerant species as well (Veneklaas and Poorter, 1998; Poorter, 1999). However, when the GRC-analysis is restricted to the three shade-intolerant species, the SLA is much more important for explaining differences in RGR at high irradiance (Figure 6B; Poorter and van der Werf, 1998). Clearly, the ecological background of species included in the comparison is important for the conclusion whether variation in assimilation rate or allocation and morphology are important for explaining interspecific variation in RGR. This is particularly an issue when shade-tolerant species grown at high irradiance are included in the comparison.

The low ULR of *Geum* at the highest irradiance is associated with a low *A*_growth_ (Figures 1B, 4A). Its *A*_sat_ was only slightly higher, thus *A*_growth_ was limited by its low photosynthetic capacity (Figure 4B, Supplement Table S3). Why do shade species often have a low photosynthetic capacity when grown at high irradiance? *Geum* is known to form only a single layer of palisade parenchyma (Pons, 1977a), which limits the development of a high *A*_sat_ (Terashima et al., 2001). A lack of the capability to develop a multilayered palisade parenchyma was also reported for *Impatiens* together with a relatively low photosynthetic capacity (Groen, 1973). Although *Impatiens* had not a particularly low *A*_sat_ in our experiment it did not increase in the highest irradiance interval (Figure 4B). A single layer of palisade parenchyma and/or a low photosynthetic capacity in high-irradiance grown shade-tolerant plants is often reported, such as in temperate herbaceous species (Osborne et al., 1994; Murchie and Horton, 1997), tropical herbaceous plants (Chow et al., 1988), tropical shrubs (Valladares et al., 2000), temperate deciduous trees (Jackson, 1967; Hanba et al., 2002) and tropical trees (Houter and Pons, 2012). Shade-intolerant species, on the other hand, generally develop multilayered palisade parenchyma at high irradiance, which is associated with their high photosynthetic capacity (Jackson, 1967; Groen, 1973; Pons, 1977a; Hanba et al., 2002). We therefore conclude that one of the likely reasons for the low photosynthetic capacity at high growth irradiance in shade-tolerant species is their incompetence to develop a multilayered palisade parenchyma.

**Figure 7:**
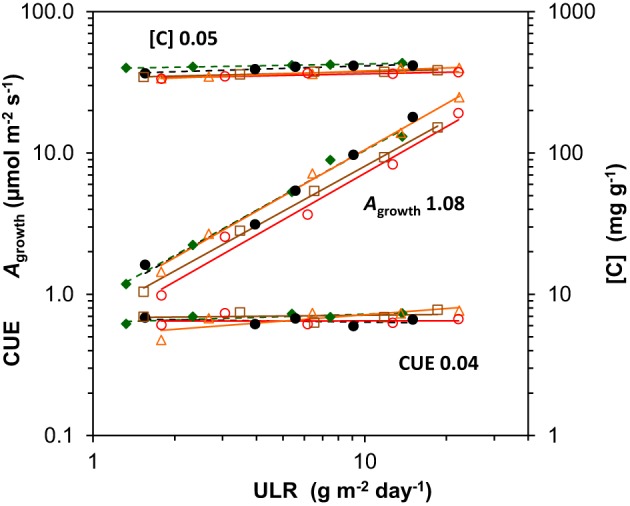
**Scaling slope analysis for each species of the contribution of its components to variation in unit leaf rate (ULR) across growth irradiances**. The components are whole-plant gross photosynthesis per unit leaf area at the growth irradiance (*A*_growth_), whole-plant C-concentration ([*C*]) and the carbon use efficiency (CUE), the fraction of daily assimilated C invested in biomass (CUE = 1 − *R/A*). The means were log-transformed and plotted against log-transformed ULR. The mean across species of the regression coefficients per component, depicting the average fractional contributions, are shown next to the regression lines. As the net assimilation rate calculated from gas exchange (NAR_ge_ = A_growth_. CUE/[*C*]) is not necessarily fully identical with ULR, the fractional contributions (*A*_growth_ and CUE positive, and [*C*] negative) do not exactly add up to unity. Species are identified by their markers and regression lines (for the legend see Figure 1).

A very high *A*_sat_ of 34 μmol m^−2^ s^−1^ at the leaf level was indeed found for *Chenopodium* grown at the highest irradiance (Figure 4B), as was reported elsewhere (Sage and Pearcy, 1987). Such a high *A*_sat_ was also reported for *Helianthus* (e.g., Fredeen et al., 1991). We found a lower value at the highest irradiance than expected, which is likely due to the rather low *N*_m_ of the leaves used for the *A*_sat_ measurements. This was not representative for the plants used for the whole-plant gas exchange (Figure 4, Supplement Tables S1–S3). The high *A*_sat_ of *Chenopodium* and supposedly also *Helianthus* facilitated the high *A*_growth_ (Figure 4A) and as *A*_growth_ is strongly related to ULR (Figure 7), also the latter. However, it should be noted that the high *A*_sat_ of the shade-intolerant species is not fully utilized in the growth conditions with a constant relatively low irradiance during daytime. *Chenopodium* and *Helianthus* had a low investment of leaf N per unit *A*_sat_ compared to *Geum*, and thus a high photosynthetic nitrogen use efficiency (PNUE_sat_; Supplement Table S3). However, as their *A*_sat_ is not fully utilized, the PNUE of the species was similar at the growth irradiance (Supplement Tables S1, S3). In field conditions, high-light exposed plants experience widely fluctuating irradiance often exceeding saturation. A high *A*_sat_ is then utilized to a much larger extent and correlates better with daily assimilation (Zotz and Winter, 1993). Larger differences in daily assimilation and consequently ULR between species with different *A*_sat_ may thus be expected at variable irradiance as in field conditions compared to the constant light regime often used in growth rooms.

### Growth at the lower irradiances

At the lower irradiances, the differences in RGR between species are more determined by differences in SLA—and to a lesser extent LMF—than by ULR (Figure 6A). Such a predominance of variation SLA for explaining species specific differences in RGR at low irradiance was more often reported (Veneklaas and Poorter, 1998; Shipley, 2006). This can be explained by the strong light limitation of photosynthesis at low irradiance resulting in a more similar *A*_growth_, which is also evident in our data (Figure 4A). A higher SLA is then of crucial importance for increased growth.

Contrary to the situation at high irradiance, we did not observe a systematic difference of RGR between shade-tolerant and shade-intolerant species at low irradiance (Figure 1A, Supplement Table S2). *Chenopodium* and *Helianthus* showed little further increase of SLA with decreasing irradiance (Figure 1C). This lack of competence to develop a high SLA at low irradiance was earlier reported for *Helianthus* (Hiroi and Monsi, 1963) and also for the shade-intolerant *Cirsium palustre* (Pons, 1977a). *Helianthus* and *Chenopodium* may thus show a stronger decrease in net C-gain when the trend continues with a further decrease in irradiance compared to species that are able to increase their SLA further.

### Tissue structure and chemistry

There was no evidence for important differences between the shade-tolerant and intolerant species in acclimation to irradiance at the chloroplast level, as derived from the chlorophyll a/b ratio and photosynthesis per unit chlorophyll (Supplement Table S3). However, at the leaf level, anatomical leaf traits are likely to make acclimation different between species, as discussed above.

Although a low LMA (and thus a high SLA) maximizes growth potential at low irradiance (Evans and Poorter, 2001), it does not necessarily increase fitness. A low LMA can weaken the leaves (Onoda et al., 2008), which may reduce leaf longevity and therefore diminish return on carbon investment (Lusk et al., 2008). A higher LMA would increase longevity when based on investment in defense components such as lignin and tannins (Lusk and Warton, 2007; Kitajima et al., 2013). However, the relatively high LMA of *Helianthus* and *Chenopodium* at low irradiance (Figure 1C) is associated with a relatively high *A*_sat_ (Figure 4B, Supplement Tables S1, S3) and thus a relatively large investment in protein-rich chloroplasts. Similar to the situation at high irradiance, the high *A*_sat_ of these species grown at low irradiance was also not utilized in the growth conditions, resulting in a low PNUE_growth_ (Supplement Tables S1, S3). Such shade leaves with a relatively high *N*_a_ (Supplement Tables S1, S3) can furthermore be attractive for herbivores. It is thus not likely that the high LMA of these species would add to their leaf longevity in shade, but rather makes them inefficient and vulnerable under these conditions.

The construction costs of plant tissue increased with irradiance (Figure 3B). This was mainly the result of lower concentrations of minerals in the dry matter, including nitrate (Supplement Table S2). The [*C*] increased for the same reason (Figure 3A). The clearly higher construction costs and [*C*] of the shade-tolerant species compared to the shade-intolerant ones (Figure 3) was not only due to lower mineral and nitrate concentrations, but also to a lower organic acid concentration in their dry matter (Supplement Table S2). These traits of the shade-tolerant species were in turn associated with high density of leaf tissue (Figure 2) and—in the case of *Geum*—also of petioles and roots (Supplement Table S1). They could, in the case of *Geum* including a low *N*_m_, be associated with better defense resulting in increased leaf longevity in shade. This is important at low irradiance, as photosynthetic rates are inherently low. The time that it takes to generate the construction costs (pay-back time) is therefore unavoidably longer (Poorter et al., 2006). The high construction costs and [*C*] of the shade-intolerant species are at the expense of RGR (equation 1), but when these traits do indeed increase longevity, they are essential for survival in shade.

### The carbon balance

The growth parameter ULR can be factorized into *A*_growth_, CUE (= 1 − *R/A*) and the [*C*] (equation 3 in Appendix 1). Using the same scaling slope analysis as explained above for the GRC, we can quantitatively asses the relative importance of variation in each of these components. The analysis shows that *A*_growth_ scaled linearly and strongly with ULR when irradiance increases, with an average slope across species close to 1.0 (Figure 7). Variation in *R/A* and [*C*] contributed little to variation in ULR (Figures 3A, 5E,F, 7). The growth calculated on the basis of gas exchange (NAR_ge_) was on average 11% higher than from harvest data (ULR). A small difference is not surprising as *A*_growth_ and *R* measured at one moment in time are not necessarily fully representative for the rates over the whole experimental period. Nevertheless, it shows that for juvenile plants grown at various light levels, ULR can be an effective estimator of photosynthesis under growth conditions.

**Figure 8:**
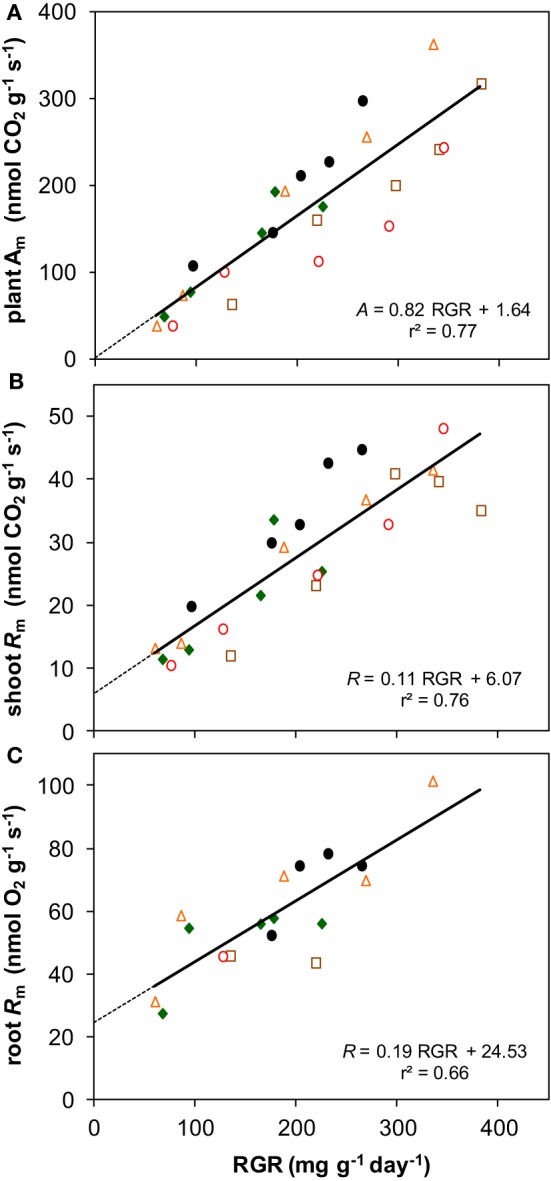
**The relationships with RGR of photosynthesis and respiration, both expressed per unit dry mass. (A)** Whole-plant *A*_m_; **(B)** shoot *R*_m_; **(C)** root *R*_m_. Means are plotted, and the equation of the regression lines and the *r*^2^ are shown.

Photosynthesis also increased proportionally with growth rate when both variables are expressed per unit dry mass (resp. *A*_m_ and RGR) (Figure 8A). Although *R*_m_ of both shoots and roots increased linearly with RGR as well, it did not scale fully proportionally with RGR as a result of the positive y-intercept (Figures 8B,C). This intercept, *R*_m_ at zero growth, is considered to be an estimate of the maintenance respiration. The part that is proportional to RGR represents the growth-related respiration (Lambers et al., 2002). From the relationships of *A*_m_ and *R*_m_ with RGR (Figure 8) it would follow that the *R/A* ratio should decrease with increasing RGR and thus with irradiance. That was indeed found for the instantaneous values as measured on shoots (Figure 5D). However, as root *R*_m_ was found to be 2.9 times higher than shoot *R*_m_ (Figures 5B,C), the decrease in RMF with decreasing irradiance (Figure 1D) has a diminishing effect on daily whole-plant *R*, which explains the relatively constant daily whole-plant *R/A* ratio across a broad range of irradiances (Figures 5E,F). Adjustments of whole-plant *R* can quickly occur when *A* changes after transfer to another irradiance (McCree and Troughton, 1966; Pons, 1977b), probably as a result of altered demand for ATP (Noguchi et al., 2001). The adjustments are apparently such that whole-plant *R/A* remained more or less constant at around 0.3 across a wide range of irradiances.

The situation is somewhat different at light levels close to the light compensation point. At 20 μmol m^−2^ s^−1^, *R/A* showed a tendency to be higher than at the other irradiances (Figure 5F). As the data set for root *R*_m_ is incomplete, we estimated *R/A* in two other ways. In the first, we assumed root *R*_m_ to be 2.9 times higher than the shoot *R*_m_, which is the average of the measurements. Shoot *R*_m_ is available for all species and irradiances and *R/A* calculated in this way thus also (Figure 5E). In the second we calculated daily whole-plant *R/A* from the linear relationship of *A*_m_, and shoot and root *R*_m_ with RGR (Figure 8), and the linear relationship of RGR and RMF with log-transformed irradiance for all species together (Supplement Figure S1). In both cases the outcome was an almost constant *R/A* with indeed increasing values below about 50 μmol m^−2^ s^−1^ (black continuous line in Figure 5F). Extrapolation of the relationships yielded an *R/A* of unity (i.e., the whole-plant light compensation point *sensu* Givnish (1988) at an irradiance of 6 μmol m^−2^ s^−1^(0.35 mol m^−2^ day^−1^). This value is higher than the measured light compensation points for growth reported by Mahmoud and Grime (1974) and Pons (1983), which were 3.2 and 1.7 μmol m^−2^ s^−1^, respectively. Our calculation assumes a constant maintenance respiration at all irradiances. The fact that lower compensation points were measured than calculated suggests that the assumption that maintenance respiration remains constant is not correct. Alternatively, maintenance respiration may be down-regulated at very low irradiances, as is also found after longer periods in darkness (Gifford, 2003).

A constant *R/A* was found for *Pisum sativum* between 100 and 400 μmol m^−2^ s^−1^ (McCree and Troughton, 1966), and *Holcus lanatus* and *Plantago major* at 150 and 300 μmol m^−2^ s^−1^ (Poorter, 2002). When calculated from shoot *A* and *R* data from Pons (1977a), assuming the same 2.9-fold higher root *R*_m_ than shoot *R*_m_, whole-plant *R/A* was also largely constant between 5 and 100% full daylight in *Geum urbanum*. In conjunction with previous observations, we therefore conclude that—with the exemption of very low light levels—the growth dependence of *R*_m_ and the decreasing RMF can establish a stable whole-plant *R/A* across a broad range of growth irradiances.

A conservative *R/A* ratio was also found when plants were grown at different temperatures in the range that plant normally encounter in their natural habitat (Gifford, 1995; Loveys et al., 2002). Whole-plant *R* and *A* acclimated to the growth temperature within this range, but higher temperatures caused an increase in *R/A* as a result of increasing *R* and decreasing *A* (Atkin et al., 2007). Elevated CO_2_ also has only marginal effects on the *R/A* ratio (Poorter, 2002; Gifford, 2003). However, a decrease in nutrient availability caused an upward shift in *R/A* (van der Werf et al., 1992; Poorter et al., 1995). This was the result of a strong increase in RMF, in combination with the fact that roots have a so much higher *R* than shoots. Hence, plants tend to maintain a homeostatic *R/A* ratio when irradiance temperature and CO_2_ vary, but *R* increases relative to *A* when nutrient availability declines as costs for nutrient acquisition increase.

### Interspecific variation in C-balance

Are there species-specific differences in the C-balance at low irradiance? And are these associated with shade-tolerance? Reliable measurements of root *R*_m_ across the whole range of irradiances are available for only two species, the shade-tolerant *Geum* and the intolerant *Helianthus* (Figure 5C). *Geum* had a more stable *R/A* than *Helianthus*, which showed a strong increase in *R/A* at the lowest irradiance (Figure 5F, Table 2). However, the available data points at low irradiance for the other species do not support a systematic difference between shade-tolerant and intolerant species (Figure 5F). The *R/A* at the shoot level shows higher values for *Helianthus* and *Chenopodium* at low irradiance (Figure 5D) and so does the whole-plant *R/A* based on shoot R_m_ measurements only (Figure 5E). The differences are significant for the shade-tolerant–shade-tolerant contrast (Table 2), but the growth analysis data do not show clear evidence of reduced net C-gain at 20 μmol m^−2^ s^−1^ for the shade-intolerant species (Figure 1A, Supplement Figure S1A). The available evidence that we have therefore does not conclusively point to a systematic difference in C-balance between our shade-tolerant and intolerant species at the lowest irradiance, which is representative for deep canopy shade. Sterck et al. (2013) arrived at the same conclusion when comparing tropical shrubs of different shade-tolerance, although they assumed a constant shoot and root *R*_m_. The number of species in our study is not large enough for broader generalizations, but when taking into account the literature data as cited above, the conclusion emerges that there is not much evidence for a systematic difference in C-balance between shade-tolerant and intolerant species at the low light levels found in deep shade under a dense canopy.

Mahmoud and Grime (1974) and Pons (1983) reduced irradiance to very low values and found no systematic difference between shade-tolerant and intolerant species in light compensation points. However, mortality was much higher for shade-intolerant species at and below the compensation point, and as far as surviving plants permitted, their RGR was estimated to be more negative. This suggests that shade-tolerant species can reduce *R* further under C-starvation than shade-intolerant species.

Many shade-intolerant species, including the ones from this study, show a pronounced shade-avoidance response at the low red: far-red ratios in canopy shade light, which involves among others increased stem and petiole growth (Morgan and Smith, 1976; Kurepin et al., 2007; Pierik et al., 2011). This is not only likely to increase *R*, but may also go at the expense of LMF (Poorter et al., 2012b), thus reducing RGR (equation 1). Shade-tolerant species, including *Geum* and to a lesser extent *Impatiens*, show a reduced shade-avoidance response in canopy shade light (Morgan and Smith, 1979; Corré, 1983b; Gommers et al., 2013). The reduced growth is likely to reduce *R*. This resembles the quiescent strategy found in submergence-tolerant species or genotypes (Bailey-Serres and Voesenek, 2008) that stop growth and reduce respiration under water where photosynthesis is negligible, as opposed to enhanced elongation growth under a negative C-balance of species that have an escape strategy such as *Rumex palustris* (Groeneveld and Voesenek, 2003). The latter shows similarities with the shade-avoidance response of shade-intolerant species, which furthermore negatively affects stem mechanics and strength (Anten et al., 2005), and can go on the expense of defense as is among others also documented for *Chenopodium* (Kurashige and Agrawal, 2005). Under natural conditions shade-intolerant species do normally not survive the lowest irradiance used in our experiment, which was equivalent to dense canopy shade. As we have little evidence for a more favorable C-balance of shade-tolerant species at the lowest irradiance under controlled conditions, tolerance to other biotic or a-biotic stresses (see discussion above) in combination with the low irradiance stress is likely to be more important for survival in canopy shade.

## Conclusions

At high irradiance, the three shade-intolerant herbaceous species used in our experiment had a higher RGR compared to the two shade-tolerant species. This was associated with a higher *A*_sat_ and consequently a higher ULR.

Daily whole-plant respiration as a fraction of gross photosynthesis (*R/A*) was essentially constant at around 0.3 over a broad range of growth irradiances. Although shoot and root *R*_m_ decreased less with decreasing irradiance than *A*_m_, the decrease in RMF in combination with the much higher root *R*_m_ compared to shoot *R*_m_ explained the constancy of whole-plant *R/A*.

At the lowest irradiance, two of the three shade-intolerant species showed a tendency of a less efficient C-balance, but there were no systematic differences in RGR, ULR or *R/A* between the shade-tolerant and shade-intolerant species. No conclusive evidence was thus found for a less favorable C-balance between the two functional groups at the lowest irradiance.

Remarkable differences between the functional groups were a higher dry matter percentage, carbon concentration and constructions costs of leaf tissue for the shade-tolerant species. These traits could be associated with better defense and therefore increased leaf longevity in shade. Superior longevity and tolerance to other stresses at low irradiance are likely to be more decisive for survival at the low irradiance in canopy shade than a superior C- balance.

### Conflict of interest statement

The authors declare that the research was conducted in the absence of any commercial or financial relationships that could be construed as a potential conflict of interest.

## Appendix 1

The relative growth rate (RGR), the increase in dry mass per unit plant mass and time, consists of three components, the unit leaf rate (ULR), the increase in dry mass per unit leaf area and time, the specific leaf area (SLA), the leaf area per leaf dry mass, and the leaf mass fraction (LMF), the leaf dry mass per plant mass (Evans, 1972).

(2) RGR=ULR.SLA.LMF

The ULR as measured from the dry mass increment per unit leaf area can also be expressed as the daily net assimilation rate based on gas exchange (NAR_ge_).

(3) $$\text{ULR}\approx {\text{NAR}}_{\text{ge}}=\left(A_{\text{growth}}-R_{\text{a}}\right)/\left[C\right]$$

(Poorter et al., 2013). *A*_growth_ is daily whole-plant gross photosynthesis and *R*_a_ is daily whole-plant respiration both expressed per unit leaf area. The molar carbon concentration in plant dry matter [*C*] converts net molar CO_2_ uptake to dry mass units. NAR_ge_ can also be calculated using the fraction of daily gross photosynthesis that is respired (*R/A*), or the carbon use efficiency (CUE), the fraction of daily gross photosynthesis that is invested in growth (CUE = 1 – *R/A*).

(4) $$A_{\text{growth}}-R_{\text{a}}=A_{\text{growth}}.\left(1-R/A\right)=A_{\text{growth}}.\text{CUE}$$
