# Supplementary material for: The effect of irradiance on the carbon balance and tissue characteristics of five herbaceous species differing in shade-tolerance
Source: Front Plant Sci. 2014 Feb 4;5:12. doi: 10.3389/fpls.2014.00012 (PMC3912841; doi:10.3389/fpls.2014.00012)
Supplement: Supplementary file 1 [file DataSheet1.PDF]

Supplemental material

T.L. Pons and H. Poorter.

The effect of irradiance on the carbon balance and tissue characteristics of five herbaceous species differing in shade-tolerance

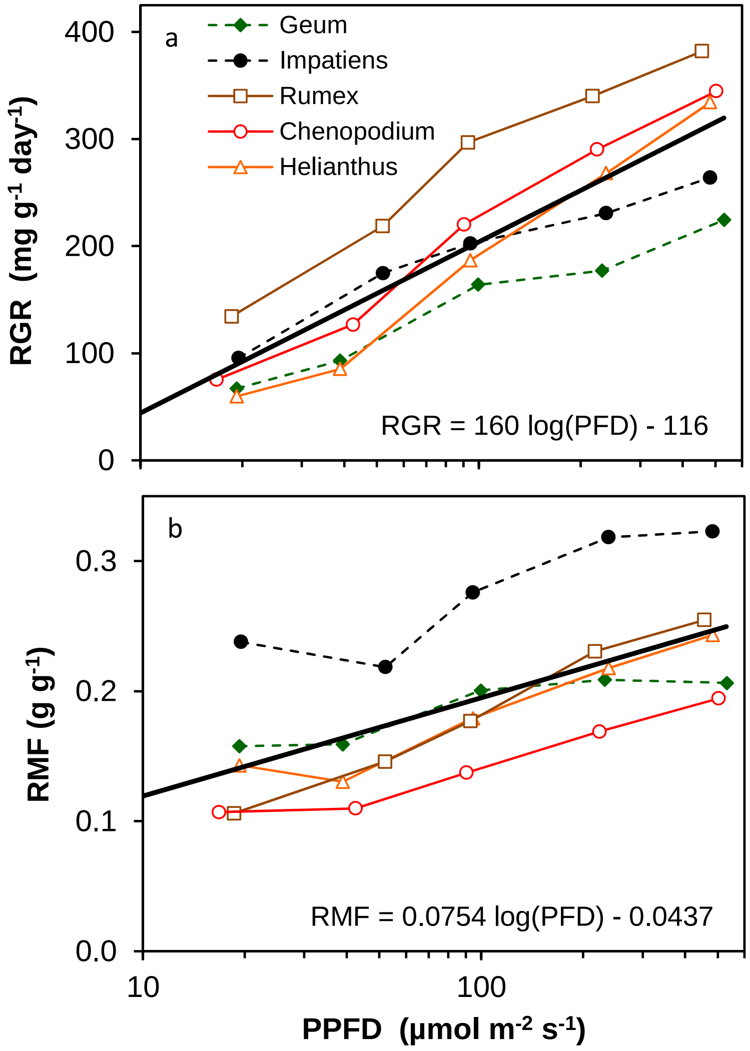

Fig. S1. Relative growth rate (RGR) and root mass fraction (RMF) plotted against irradiance (PFD; log-scale). Data are the same as in Fig. 1a & d. Common regression lines are drawn. The equations were used for the calculation of the irradiance dependence of the daily *R/A* ratio as depicted in Fig. 5f.

Supplement Table S1. Leaf area ratio (LAR [= SLA x LMF]), stem mass fraction (SMF), leaf mass per unit area (LMA [= 1/SLA]), organic nitrogen per unit leaf area (Na),photosynthetic nitrogen use efficiency (PNUE) or the rate of photosynthesis expressed per unit leaf nitrogen at the growth irradiance and the dry matter percentages of leaves, stems and roots.
Means are shown with standard deviations (sd) in smaller font (n=8). Na (n=2) was calculated from Nm (Table S2) and LMA.
For the two-way ANOVA are shown the adjuste r^2 for the model, the total df, and the percentages explained variance of total explained variance for the effects of Species, irradiance (PFD) and their interaction. In addition are shown the explained variance of the a-priori contrasts of shade-tolerant (Tol) vs shade-intolerant (Intol) species for the Species main effect and the Species X PFD interaction.
The percentages refer to explained variance relative to the explained variance of the Species and Species x PFD effect respectively.
Significance levels are: ns, P<0.05; +, P<0.1; *, P<0.05**, P<0.01, ***, P<0.001.

Species	PFD	LAR	SMF	LMA	N_a	PNUE	Dry Matter Percentage (% of fresh mass)		
	$\mu mol\ m^{-2}\ s^{-1}$	($m^2\ kg^{-1}$)	($g\ g^{-1}$)	($g\ m^{-2}$)	($mmol\ m^{-2}$)	($mmol\ mol^{-1}\ s^{-1}$)	Leaf	Stem	Root
Geum	19	50.61 2.01	0.21 0.01	12.61 0.50	30.9 0.6	31.6 0.2	13.35 0.53	8.82 0.57	9.44 0.66
	39	41.74 6.34	0.20 0.04	15.74 2.49	42.1 0.2	46.7 5.4	14.90 1.37	9.13 1.05	8.47 0.38
	100	30.17 2.15	0.15 0.01	21.97 1.86	57.6 2.0	82.0 4.4	17.88 0.58	11.26 0.31	8.23 0.48
	232	23.97 2.35	0.14 0.01	27.64 2.59	63.6 1.7	123.2 5.1	20.46 0.83	12.98 0.40	8.39 0.67
	532	16.52 2.13	0.13 0.01	41.20 6.01	92.9 0.1	125.6 5.7	24.71 1.79	15.51 1.36	8.71 0.62
Impatiens	20	61.18 4.72	0.22 0.01	9.64 0.75	30.8 1.6	45.3 3.2	6.43 0.28	3.23 0.24	4.77 1.47
	52	45.47 6.69	0.21 0.02	14.13 3.15	42.9 0.3	62.2 3.3	8.21 0.77	3.59 0.19	4.07 0.64
	95	38.27 8.19	0.16 0.01	16.30 4.10	61.2 0.6	79.0 1.4	8.75 1.09	4.91 0.95	4.63 0.92
	238	25.31 1.73	0.13 0.01	22.25 1.61	95.5 0.3	90.0 1.2	11.35 0.47	5.73 0.16	4.13 0.78
	483	17.82 2.16	0.12 0.01	32.38 4.47	135.0 7.6	121.0 0.5	13.58 0.50	6.33 0.37	4.19 0.63
Rumex	19	87.65 4.09	0.15 0.00	8.51 0.39	25.8 0.8	34.3 3.3	4.64 0.06	4.77 0.18	5.75 0.45
	52	63.34 3.39	0.14 0.01	11.49 0.74	39.1 2.1	66.8 12.3	5.48 0.22	5.31 0.22	5.81 0.62
	93	45.84 3.66	0.12 0.01	15.49 1.14	56.0 2.2	81.9 9.4	6.20 0.27	6.35 0.34	5.07 0.29
	217	28.84 1.75	0.11 0.00	22.97 1.26	84.0 1.4	98.8 13.2	7.25 0.41	7.68 0.50	5.05 0.47
	457	20.67 2.06	0.10 0.00	32.24 2.28	121.3 1.1	115.0 20.3	8.45 0.36	8.84 0.32	5.16 0.35
Chenopodium	17	42.69 5.11	0.29 0.03	14.31 1.30	49.5 0.1	16.9 3.8	7.10 0.39	7.01 0.77	5.84 0.26
	43	41.11 3.07	0.27 0.04	15.02 0.59	55.6 1.6	43.7 2.6	7.24 0.12	7.09 0.61	5.38 0.48
	91	35.90 2.16	0.23 0.03	17.84 1.19	69.5 3.9	43.6 9.6	8.13 0.11	8.15 0.19	5.57 0.75
	224	22.82 2.02	0.24 0.04	26.34 1.32	106.9 3.6	66.5 0.1	9.39 0.31	9.30 0.23	5.90 0.34
	504	15.57 2.11	0.23 0.04	37.52 3.34	158.2 6.6	106.7 15.8	11.18 0.43	10.54 0.32	6.83 0.61
Helianthus	19	33.42 1.48	0.34 0.03	15.69 1.07	51.0 2.9	21.9 2.2	6.66 0.23	3.89 0.10	4.51 0.50
	39	32.35 2.83	0.36 0.03	15.87 1.39	55.6 3.0	41.6 8.1	7.68 0.28	4.45 0.34	4.84 0.31
	95	29.17 1.29	0.24 0.02	19.96 0.66	62.9 4.8	101.3 7.7	7.93 0.63	5.45 0.32	5.35 0.44
	238	19.77 0.77	0.21 0.01	28.89 0.85	110.1 2.3	112.8 3.2	9.81 0.68	6.81 0.23	5.54 0.54
	483	15.04 0.46	0.19 0.02	37.69 1.07	136.2 0.1	167.3 6.1	10.77 0.96	7.34 0.46	5.67 0.46
Two-way ANOVA									
Species		17 ***	67 ***	9 ***	11 ***	9 ***	73 ***	67 ***	92 ***
PFD		80 ***	29 ***	88 ***	86 ***	81 ***	26 ***	32 ***	1 +
Species x PFD		4 ***	5 ***	3 ***	4 ***	9 ***	1 ***	1 ***	7 ***
Tol - Intol		0 ns	6 ***	0 ns	32 ***	21 ***	55 ***	2 ***	5 ***
Tol - Intol x PFD		16 **	10 ***	21 ***	33 ***	23 ***	45 ***	15 *	25 **
total df		199	199	199	49	49	199	199	199
r^2		0.95	0.93	0.94	0.99	0.96	0.98	0.97	0.82

Supplement Table S2. Chemical composition of dry matter in leaves, and stem and roots combined, and the calculated construction costs (g glucose (g dry matter) $^{-1}$). Minerals are including nitrate.
Samples from the three harvests were pooled as explained in Materials and Methods. Means are shown with standard deviations (sd) in smaller font (n=2).
For the two-way ANOVA are shown the adjuste r^2 for the model, the total df, and the percentages explained variance of total explained variance for the effects of Species, irradiance (PFD) and their interaction.
In addition are shown the explained variance of the a-priori contrasts of shade-tolerant (Tol) vs shade-intolerant (Intol) species for the Species main effect and the Species X PFD interaction.
The percentages refer to explained variance relative to the explained variance of the Species and Species x PFD effect respectively. Significance levels are: ns, P<0.05; +, P<0.1; *, P<0.05**, P<0.01, ***, P<0.001.

Species	PFD	Nitrate (mg g^{-1})		Minerals (mg g^{-1})		Organic Acids (mg g^{-1})		Organic Nitrogen (mg g^{-1})		Carbon concentration (mg g^{-1})		Construction Costs (g g^{-1})		
	µmol m^{-2} s^{-1}	Leaf	Stem + Root	Leaf	Stem + Root	Leaf	Stem + Root	Leaf	Stem + Root	Leaf	Stem + Root	Leaf	Stem + Root	Plant
Geum	19	89.5 9.4	96.0 3.8	153.1 9.8	216.4 6.6	58.2 3.4	31.0 2.8	33.3 1.8	20.1 3.1	410 4	383 8	1.38 0.02	1.23 0.03	1.33 0.03
	39	81.2 2.3	95.2 1.3	141.4 2.1	202.8 2.1	62.6 8.8	21.7 17.7	35.3 0.1	22.0 0.9	416 3	388 1	1.41 0.02	1.26 0.00	1.36 0.01
	100	72.4 3.6	74.8 6.8	125.0 3.3	179.3 7.5	60.0 10.9	31.3 2.8	35.3 0.4	26.0 2.4	426 1	397 3	1.44 0.01	1.30 0.02	1.39 0.01
	232	64.6 3.7	68.9 4.7	113.1 4.4	177.1 17.3	56.2 11.5	35.0 20.4	31.1 0.4	27.0 0.3	432 3	403 6	1.44 0.01	1.33 0.01	1.40 0.00
	532	51.3 1.3	70.1 1.4	89.7 4.1	158.8 10.8	60.4 14.1	24.5 9.1	29.4 0.4	26.3 1.6	440 1	415 1	1.44 0.00	1.37 0.02	1.42 0.00
Impatiens	20	36.6 5.9	79.0 6.3	138.9 0.4	331.1 12.2	90.0 11.1	109.5 6.3	53.7 1.3	35.6 0.4	435 5	281 22	1.60 0.02	0.92 0.10	1.29 0.04
	52	26.6 1.4	106.7 44.1	123.1 11.3	277.0 60.2	97.5 8.6	84.9 43.4	56.5 0.8	31.6 10.1	439 0	334 8	1.62 0.02	1.11 0.05	1.39 0.03
	95	20.4 3.2	107.1 4.3	106.7 3.2	260.5 18.4	96.3 1.1	36.7 6.7	61.1 0.1	27.7 2.1	444 3	358 13	1.65 0.01	1.20 0.06	1.46 0.02
	238	16.7 1.3	81.8 3.6	97.0 4.7	238.5 11.3	91.4 2.4	57.8 11.2	62.2 1.5	35.0 0.8	448 2	371 6	1.67 0.01	1.28 0.01	1.50 0.00
	483	12.1 0.1	66.4 18.3	92.8 1.1	213.4 22.8	89.4 9.1	64.6 21.5	60.3 0.6	38.2 8.3	445 3	379 8	1.64 0.02	1.31 0.02	1.49 0.02
Rumex	19	61.5 9.7	72.6 14.0	215.6 10.3	231.4 17.4	139.7 15.1	79.1 8.7	42.5 1.5	31.6 4.0	349 2	328 3	1.19 0.01	1.03 0.02	1.15 0.01
	52	42.4 2.7	82.3 6.3	187.5 0.3	221.7 3.4	163.6 3.8	72.2 2.2	45.0 1.2	31.8 0.6	361 8	354 6	1.23 0.05	1.16 0.02	1.21 0.03
	93	18.5 1.7	70.8 0.7	129.7 1.4	175.3 4.0	166.0 3.7	73.8 0.5	47.8 0.8	32.7 1.7	379 11	371 9	1.28 0.06	1.20 0.05	1.25 0.06
	217	22.6 4.2	70.1 4.1	143.7 2.1	179.9 10.6	171.1 1.5	64.0 17.2	50.7 0.5	34.8 1.3	377 5	373 7	1.30 0.02	1.22 0.03	1.27 0.03
	457	11.8 3.3	66.9 7.3	124.6 4.1	170.3 6.6	164.2 7.5	66.0 1.8	49.2 0.7	36.1 1.7	384 5	384 4	1.30 0.03	1.28 0.02	1.30 0.02
Chenopodium	17	67.8 2.7	98.0 1.7	189.4 5.5	256.0 4.6	181.5 10.5	125.9 2.6	47.4 0.2	35.1 0.9	354 6	309 12	1.21 0.04	0.98 0.06	1.11 0.05
	43	42.2 1.2	99.9 24.1	165.2 1.9	242.3 12.1	207.9 5.9	121.8 8.8	52.1 0.6	26.9 4.4	366 10	323 4	1.27 0.05	1.00 0.01	1.16 0.04
	91	16.1 2.4	81.9 2.7	139.3 5.0	215.4 8.7	222.8 5.8	125.7 14.2	55.8 0.9	31.2 2.1	383 8	342 12	1.35 0.04	1.09 0.04	1.24 0.04
	224	13.9 0.8	77.8 4.6	129.9 6.7	204.2 1.8	232.4 0.7	116.8 11.2	55.2 1.3	31.1 2.4	370 6	354 3	1.27 0.04	1.14 0.02	1.21 0.04
	504	7.2 1.1	50.7 1.4	115.8 0.3	158.8 3.0	235.6 12.0	135.9 5.6	54.5 1.2	32.8 2.6	377 7	367 9	1.29 0.04	1.17 0.06	1.23 0.05
Helianthus	19	87.0 7.0	83.6 3.1	214.3 9.4	286.6 1.5	116.1 24.4	118.0 18.9	46.7 2.5	37.5 0.4	389 7	283 4	1.41 0.03	0.89 0.02	1.16 0.02
	39	89.3 7.1	85.9 11.4	215.9 11.8	249.8 42.1	142.5 31.2	125.2 42.5	48.4 1.6	32.6 0.9	385 1	307 38	1.40 0.01	0.95 0.15	1.18 0.07
	95	90.3 23.4	63.1 1.5	234.1 43.7	229.8 17.1	114.9 74.5	148.2 44.6	44.1 5.1	36.5 1.1	384 2	331 2	1.39 0.03	1.08 0.02	1.26 0.01
	238	37.2 1.6	79.6 2.1	136.4 8.6	209.0 6.0	147.3 15.3	55.5 12.8	51.6 2.6	26.3 0.1	413 5	380 7	1.47 0.03	1.25 0.03	1.37 0.00
	483	19.7 0.8	75.5 10.6	125.1 4.0	209.7 20.8	161.4 6.6	57.8 0.9	49.4 0.7	27.2 2.4	415 1	379 6	1.46 0.00	1.25 0.02	1.37 0.01
Two-way ANOVA														
Species		45 ***	11 ns	42 ***	45 ***	96 ***	79 ***	91 ***	55 ***	87 ***	38 ***	93 ***	35 ***	65 ***
PFD		43 ***	48 ***	48 ***	49 ***	2 ns	4 ns	3 ***	5 ns	9 ***	49 ***	5 ***	52 ***	29 ***
Species x PFD		12 ***	41 *	10 ***	5 ns	3 ns	17 +	6 ***	40 **	4 **	13 **	3 +	13 *	6 +
Tol - Intol		4 ***	56 *	82 ***	2 ns	78 ***	58 ***	8 ***	26 ***	84 ***	28 ***	57 ***	50 ***	90 ***
Tol - Intol x PFD		47 ***	12 ns	4 ns	5 ns	44 ns	25 ns	31 ***	27 *	10 ns	6 ns	10 ns	6 ns	10 ns
total df		49	49	49	49	49	49	49	49	49	49	49	49	49
r^2		0.97	0.54	0.96	0.84	0.86	0.77	0.97	0.66	0.97	0.89	0.95	0.86	0.92

Supplement Table S3. Trait values of recently matured leaves used for the gas exchange measurements. Leaf mass per area (LMA), chlorophyll per area, chlorophyll a/b ratio organic nitrogen per unit dry mass (N_m) and per unit leaf area (N_a), dark respiration per dry mass (R_m), photosynthesis at the growth irradiance per unit leaf area (A_{growth}) and per organic nitrogen ($PNUE_{growth}$), light saturated rate of photosynthesis per unit leaf area (A_{sat}), per chlorophyll (A_{chl}) and per organic nitrogen ($PNUE_{sat}$).
Means (n=3) are shown with standard deviation (sd) in smaller font. For the two-way ANOVA are shown, adjusted r2 of the model, total df, and percentages explained variance of total explained variance for the effects of Species and irradiance (PFD), and their interaction. Significance levels are: ns, P<0.05; **, P<0.01, ***, P<0.001.

Species	PFD $\mu mol\ m^{-2}\ s^{-1}$	LMA $g\ m^{-2}$	chlorophyll $\mu mol\ m^{-2}$	chlorophyll a/b $mol\ mol^{-1}$	N_m $mg\ g^{-1}$	N_a $mmol\ m^{-2}$	R_m $nmol\ g^{-1}\ s^{-1}$	A_{growth} $\mu mol\ m^{-2}\ s^{-1}$	A_{sat} $\mu mol\ m^{-2}\ s^{-1}$	A_{chl} $mmol\ mol^{-1}\ s^{-1}$	PNUEgrowh $mmol\ mol^{-1}\ s^{-1}$	$PNUE_{sat}$ $mmol\ mol^{-1}\ s^{-1}$
Geum	95	22.4 2.1	380 58	3.83 0.07	34.5 1.5	55.2 7.6	21.2 1.9	4.42 0.13	8.61 0.75	22.9 1.8	81 8	157 13
	238	36.4 4.4	366 21	4.01 0.20	30.4 1.3	78.9 7.0	22.1 3.4	8.63 0.53	11.53 1.09	31.5 1.6	110 5	146 6
	483	42.1 5.2	250 36	4.69 0.27	29.4 2.0	87.9 5.3	19.8 2.1	11.84 0.53	13.01 0.40	52.8 7.3	135 2	148 5
Impatiens	20	7.9 0.7	279 66	3.15 0.16	49.2 3.9	27.7 0.4	22.8 1.4	1.08 0.09	4.14 0.68	15.0 1.3	39 3	149 23
	52	10.7 0.9	358 11	3.47 0.08	54.4 2.3	41.6 5.0	26.8 1.5	2.88 0.13	7.90 0.99	22.0 2.1	70 9	190 3
	95	14.6 0.1	389 17	3.73 0.03	52.6 1.8	54.8 2.0	36.2 2.6	5.48 0.32	12.87 0.58	33.1 2.0	100 7	235 11
	238	28.9 2.7	441 36	4.41 0.09	54.2 1.6	111.8 13.4	40.8 4.4	14.55 2.61	27.02 4.36	62.0 14.2	130 11	241 12
	483	31.0 2.5	334 30	5.64 0.09	52.9 2.4	117.3 14.7	52.1 6.5	18.22 1.24	25.47 3.04	76.2 3.0	156 16	218 24
Rumex	20	7.5 0.3	244 8	3.47 0.04	45.0 1.7	24.2 0.8	19.3 5.6	0.96 0.12	3.58 0.49	14.7 2.4	40 5	147 16
	55	9.7 1.0	283 29	3.64 0.04	44.1 1.5	30.6 3.5	24.9 3.5	2.56 0.25	5.98 1.16	21.0 1.8	84 10	194 16
	95	13.0 0.5	319 12	3.97 0.05	48.5 3.6	45.2 4.7	35.1 5.3	5.68 0.32	11.02 0.27	34.5 1.6	127 19	245 20
	220	22.0 1.0	389 17	4.35 0.10	45.5 1.4	71.4 2.2	38.9 1.7	9.90 0.40	17.09 0.84	44.0 1.2	139 2	239 4
	470	33.9 1.5	426 14	4.62 0.11	40.2 0.8	97.3 4.0	38.9 1.1	17.01 1.00	24.11 0.73	56.7 3.6	175 14	248 13
Chenopodium	20	10.6 0.8	355 31	3.98 0.11	57.6 10.8	43.0 4.6	35.0 15.3	0.92 0.24	6.33 0.59	17.9 1.5	22 7	149 29
	55	14.2 0.8	467 33	4.03 0.04	56.3 4.5	56.8 1.3	35.9 2.3	3.50 0.18	10.62 0.67	22.8 0.6	62 4	187 15
	95	20.1 1.8	523 37	4.25 0.13	53.5 1.6	76.5 4.7	50.0 18.2	6.35 1.47	19.09 3.76	36.4 6.2	84 25	252 66
	220	26.5 1.4	507 67	4.50 0.09	51.4 1.0	97.6 5.1	50.3 8.8	11.99 0.93	24.41 1.52	48.5 3.7	124 20	259 7
	470	38.5 2.0	478 34	5.43 0.26	51.8 2.9	145.7 2.0	57.1 3.4	21.01 0.82	35.19 0.94	73.8 4.5	141 1	243 12
Helianthus	20	12.0 1.0	360 29	3.52 0.08	55.9 2.7	47.8 6.2	26.3 3.1	0.93 0.07	7.45 0.91	20.6 1.1	20 4	156 10
	52	16.3 0.7	536 82	3.69 0.02	57.3 1.1	66.7 3.5	40.4 6.7	2.94 0.34	15.07 0.74	28.4 3.5	44 7	226 16
	90	18.7 0.8	507 41	3.81 0.11	57.4 1.5	76.8 4.9	41.2 3.2	5.02 0.58	19.81 1.24	39.1 1.4	66 11	259 22
	202	24.4 0.8	495 32	4.18 0.13	55.2 1.3	96.0 2.9	48.1 3.1	10.06 2.02	28.01 2.29	56.9 7.3	105 22	291 15
	457	33.8 3.6	524 72	4.52 0.04	43.8 5.2	105.1 6.9	43.0 2.1	20.84 0.36	27.57 1.02	53.2 6.6	199 14	263 19
Two-way ANOVA												
Species		10 ***	34 ***	11 ***	87 ***	17 ***	57 ***	1 ***	21 ***	8 ***	12 ***	45 ***
PFD		86 ***	31 ***	77 ***	6 ***	77 ***	38 ***	98 ***	76 ***	88 ***	86 ***	54 ***
Species x PFD		4 ***	15 ***	11 ***	7 **	6 ***	5 ns	1 **	3 ***	4 ***	2 ns	2 ns
total df		68	68	68	66	66	68	68	68	68	66	66
r^2		0.98	0.83	0.96	0.97	0.97	0.79	0.99	0.97	0.96	0.94	0.84
